# Adverse event profile of five anti head and neck squamous cell carcinoma drugs: a descriptive analysis from WHO-VigiAccess

**DOI:** 10.3389/fphar.2025.1602276

**Published:** 2025-06-24

**Authors:** Weimin Gao, Zhigang Xia, Tingfeng Zhou, Youlin Dong

**Affiliations:** ^1^ Department of Otolaryngology, The Second Affiliated Hospital of Jiaxing University, Jiaxing, China; ^2^ Department of Otolaryngology, The First Affiliated Hospital of Wenzhou Medical University, Wenzhou, China

**Keywords:** HNSCC, WHO-vigiaccess, anti-HNSCC drugs, adverse drug reactions, disproportionality analysis

## Abstract

**Background:**

Head and neck squamous cell carcinoma (HNSCC) remains a significant global health concern, with treatment outcomes for advanced or metastatic stages being suboptimal despite the availability of various targeted therapies and immunotherapies. This study evaluates five FDA-approved anti-HNSCC drugs—cetuximab, pembrolizumab, nivolumab, atezolizumab, and durvalumab—focusing on the adverse drug reactions (ADRs) associated with their use as reported in the WHO VigiAccess database.

**Methods:**

A retrospective analysis was conducted on ADR reports from the WHO-VigiAccess database, focusing on demographic information (age, gender, and geographical distribution) and ADR classification. The disproportionality analysis was used to identify ADRs through Reporting Odds Ratios (ROR) and Proportional Reporting Ratios (PRR). ADRs were categorized into 27 system organ classes (SOCs) for comparison across the five drugs.

**Results:**

A total of 145,678 ADR reports were analyzed. Cetuximab exhibited the highest incidence of skin and subcutaneous tissue disorders (20.88%), while durvalumab showed elevated respiratory system disorders (18.53%). Pembrolizumab and nivolumab had notable immune-related adverse events, with malignant neoplasm progression reported at 5.56% and 4.23%, respectively. Atezolizumab was primarily associated with blood and lymphatic system disorders (5.51%). Disproportionality analysis revealed significant safety concerns for each drug, such as skin toxicity for cetuximab, respiratory complications for durvalumab, and reproductive system risks for nivolumab.

**Conclusion:**

This comparative pharmacovigilance study highlights the diverse safety profiles of the five anti-HNSCC drugs. Clinicians should consider these ADRs when treating patients, especially elderly individuals or those with comorbidities. Personalized monitoring strategies should be developed to minimize risks and optimize therapeutic outcomes for HNSCC patients.

## 1 Introduction

Head and neck squamous cell carcinoma (HNSCC) is a malignant tumor originating from the mucosal epithelium of the oral cavity, pharynx, larynx, and other regions of the upper digestive tract (Jiang et al., 2025). With an annual incidence exceeding 600,000 cases worldwide, HNSCC demonstrates significant geographical variations in disease prevalence (Wang and Anderson, 2022). HNSCC remains a major global health concern, with an estimated 946,456 new cases and 482,001 deaths reported annually worldwide (Bray et al., 2024). Despite advancements in diagnostic imaging and multimodal therapies, the 5-year survival rate for HNSCC remains relatively low, with fewer than 50% of patients surviving beyond this period (Ferlay et al., 2019). The incidence is generally higher in males than in females, likely due to higher rates of tobacco and alcohol consumption among men (Jiang et al., 2024). A similar sex-based disparity is observed in mortality rates. The treatment of HNSCC typically requires multidisciplinary comprehensive therapy, including surgery, radiotherapy, and chemotherapy (Cao et al., 2024). These treatment methods not only cause physical suffering to patients but also impose a heavy economic burden. The direct medical costs associated with HNSCC include hospitalization, surgery, chemoradiotherapy, and various diagnostic procedures. Indirect costs arise from productivity loss due to illness and treatment, as well as caregiving-related expenses borne by family members (Haddad et al., 2019). Moreover, the high recurrence rate of HNSCC further increases treatment complexity and economic burden (Umbreit et al., 2016). A deeper understanding of the therapeutic landscape, associated adverse events is therefore essential to guide clinical decision-making and improve outcomes in HNSCC management.

In the treatment landscape of HNSCC, five major systemic agents—cetuximab, pembrolizumab, nivolumab, atezolizumab, and durvalumab—have received clinical approval based on their demonstrated efficacy and safety profiles. These drugs fall into two main therapeutic categories: targeted therapy and immune checkpoint inhibitors. Cetuximab, a monoclonal antibody against the epidermal growth factor receptor (EGFR), has historically played a key role in the EXTREME regimen (cetuximab + platinum + 5-fluorouracil), which was considered the first-line standard of care for recurrent/metastatic (R/M) HNSCC prior to the introduction of immunotherapy (Vasiliadou et al., 2021). The standard cetuximab dosing protocol consists of an initial loading dose of 400 mg/m^2^ followed by 250 mg/m^2^ weekly (Chen et al., 2013). With the advent of immune checkpoint inhibitors, particularly anti-PD-1 antibodies, treatment strategies have evolved significantly. Pembrolizumab, as demonstrated in the KEYNOTE-048 trial, has become a first-line standard for R/M HNSCC either as monotherapy in patients with PD-L1 Combined Positive Score ≥1, or combination with platinum-based chemotherapy in those with more aggressive disease (Fan et al., 2020). The recommended dosage of pembrolizumab is either 200 mg every 3 weeks or 400 mg every 6 weeks (Haas et al., 2023). Nivolumab is approved for patients with R/M HNSCC who experience disease progression on or after platinum-based therapy, typically administered at 240 mg every 2 weeks or 480 mg every 4 weeks (Cohen et al., 2019). Although atezolizumab and durvalumab are not yet standard treatments for HNSCC, they have received regulatory approval in other solid tumors such as non-small cell lung cancer and urothelial carcinoma, and are currently being explored in head and neck cancers through ongoing clinical trials (Sodji et al., 2017). Atezolizumab is typically dosed at 1200 mg every 3 weeks, while durvalumab is administered at 10 mg/kg every 2 weeks (Prelaj et al., 2022). Treatment decisions for HNSCC are influenced by a variety of clinical and demographic factors, including patient age, performance status, comorbidities, and prior treatment history (Klinghammer et al., 2022). Understanding the mechanism of action, approved indications, dosing regimens, and real-world application of these agents is essential for optimizing individualized treatment strategies.

The utilization of real-world data (RWD) and spontaneous reporting systems (SRS) constitutes a validated approach for pharmacovigilance assessment (Jo et al., 2021). Since the 1960s, SRS has served as the cornerstone of pharmacovigilance, enabling early detection of adverse drug reactions and population-level safety evaluation (Srba et al., 2012). The WHO Collaborating Centre for International Drug Monitoring (Uppsala Monitoring Centre) maintains a global adverse drug reactions (ADRs) database critical for comparative drug safety analytics (Shetti et al., 2011). These data repositories play pivotal roles in enhancing HNSCC drug safety profiles and refining therapeutic protocols. Expanded therapeutic applications necessitate intensified safety surveillance.

This study evaluates five FDA-approved anti-HNSCC agents: cetuximab, pembrolizumab, nivolumab, atezolizumab, and durvalumab. These therapeutics demonstrate validated efficacy in advanced/recurrent HNSCC through multicenter clinical trials. However, treatment tolerance diminishes in elderly patients due to tumor progression, physiological decline, and immunosenescence (Song et al., 2024). Age-related pharmacodynamic alterations increase vulnerability to immunotherapy/targeted therapy toxicities, exacerbated by tumor heterogeneity and therapeutic complexity (Su et al., 2023). Geriatric treatment disparities manifest as reduced therapeutic response, amplified adverse effects, and compromised disease management (Schupack et al., 2022). Therapeutic efficacy in advanced disease is constrained by immune evasion mechanisms, tumor microenvironment dynamics, and patient performance status. This necessitates personalized therapeutic regimens tailored to individual patient profiles. We conducted a descriptive analysis of the spontaneously reported adverse events recorded in the VigiAccess database, aiming to compare the differences in adverse reactions associated with the five anti-HNSCC drugs. By analyzing the types and frequencies of adverse events, we sought to identify key safety concerns that may impact drug use, providing valuable insights for future clinical practice.

## 2 Methods

### 2.1 Drug samples

This study analyzes five therapeutic agents for HNSCC: cetuximab, pembrolizumab, nivolumab, atezolizumab, and durvalumab. Selection criteria (Table 1) prioritized clinical utility in HNSCC management and mechanistic targeting of immune evasion pathways. This study selected five anti-HNSCC drugs for analysis based on the following considerations: (1) Widespread Clinical Adoption: These agents are among the most commonly used drugs in clinical practice for HNSCC treatment, particularly for recurrent or metastatic HNSCC (Goel et al., 2022; Taberna et al., 2019). (2) Representative Mechanisms of Action: These drugs exemplify the two primary therapeutic strategies for HNSCC—EGFR inhibition and PD-1/PD-L1 immune checkpoint blockade (Wang et al., 2024). Their inclusion provides a comprehensive overview of ADR profiles associated with current HNSCC treatment paradigms. (3) Guideline Recommendations: These agents are recommended for HNSCC treatment in authoritative guidelines such as the NCCN (Cohen et al., 2019). (4) Clinical Trial Evidence: Robust clinical trial data support the use of these drugs in HNSCC management, establishing a solid foundation for this study (Yao et al., 2025). (5) Data Accessibility: Selection of these drugs ensures sufficient sample size within the VigiBase database, enhancing the reliability of study findings.

**TABLE 1:** Overview of five anti-HNSCC drugs.

Drug Name	Structure	Target	Indications	First Marketed year
Cetuximab	Monoclonal antibody (IgG1, chimeric)	EGFR	HNSCC, Metastatic colorectal cancer, Squamous cell carcinoma of head and neck	2004
Pembrolizumab	Monoclonal antibody (IgG4, humanized)	PD-1	HNSCC, Melanoma, Non-small cell lung cancer (NSCLC), Hodgkin lymphoma, Gastric cancer	2014
Nivolumab	Monoclonal antibody (IgG4, fully human)	PD-1	HNSCC, Melanoma, NSCLC, Renal cell carcinoma, Hodgkin lymphoma	2014
Atezolizumab	Monoclonal antibody (IgG1, humanized)	PD-L1	HNSCC, Urothelial carcinoma, NSCLC, Triple-negative breast cancer	2016
Durvalumab	Monoclonal antibody (IgG1, human)	PD-L1	HNSCC, Locally advanced or metastatic urothelial carcinoma, NSCLC	2017

### 2.2 Search strategy and data source

The WHO-VigiAccess database was queried in March 2025 for adverse event reports associated with HNSCC immunotherapies. Accessible via https://www.vigiaccess.org, the platform provides aggregated global data including demographic parameters (age, gender) and geographical distributions. The Uppsala Monitoring Centre (UMC) maintains this pharmacovigilance data through its WHO Programme for International Drug Monitoring (PIDM) portal (Hussain et al., 2021). VigiAccess interfaces with VigiBase - the world’s largest pharmacovigilance database established in 1968, initially comprising 10 participating nations. By March 2022, VigiBase encompassed 155 full members and 21 associate members under PIDM. Member states submit validated Individual Case Safety Reports (ICSRs) from healthcare professionals, patients, and manufacturers through national regulatory agencies (Ke et al., 2024). Toxicity profiles were characterized using MedDRA classification (System Organ Class [SOC] and Preferred Term [PT]) for adverse event categorization. The analysis focused on 27 symptom-relevant SOCs and PT-level frequency patterns for each agent’s ADRs. Severity stratification utilized outcome codes: fatal outcomes, hospitalization-requiring events, and life-threatening incidents. Agent-specific search filters ensured precise data extraction. WHO-VigiAccess enhances pharmacovigilance research through transparent global ADR data sharing.

### 2.3 Disproportionality analysis

This study implemented disproportionality analysis using the Reporting Odds Ratio (ROR) and Proportional Reporting Ratio (PRR) to evaluate immunotherapy-associated adverse events (AEs) in HNSCC treatment (Rothman et al., 2004; Evans et al., 2001). These quantitative methods are standard pharmacovigilance tools for AE signal detection. ROR quantifies the probability of disproportionate reporting (PDRAE) for specific drug-AE combinations relative to comparator medications (Rahman et al., 2017). The algorithm incorporates four contingency table elements: a (target drug-AE pairs), b (target drug non-AE reports), c (non-target drug AE reports), and d (non-target drug non-AE reports). Minimum case requirement (a≥5) ensures statistical stability in ROR computation. Significant disproportionality signals were defined as ROR>2. The formula provides the ROR.:

$$\text{ROR}=\frac{a/c}{b/d}$$

PRR provides a complementary assessment of reporting imbalance through incidence ratio comparison. PRR analysis applied an equivalent case threshold (≥5 reports) for validity. PRR≥2 with χ^2^ ≥ 4 (equivalent to p < 0.05) and ≥3 cases defined statistically significant signals. These thresholds minimize false-positive signals from random reporting variation. Dual-methodology analysis enabled robust detection of disproportionate AE patterns across five HNSCC immunotherapeutics. The generated safety signals contribute essential pharmacovigilance intelligence for risk mitigation strategies. The formula provides the PRR.:

$$\text{PRR}=\frac{a/\left(a+b\right)}{c/\left(c+d\right)}$$

### 2.4 Statistical analysis

This study adopts a retrospective quantitative research method, exploring past situations by analyzing current results. We used Excel to analyze the gender, age, and regional characteristics of victims of ADR from five anti-HNSSC drugs. The data sources include current status, case reports, case series, etc. The ADR reporting rate for each drug is defined by dividing the number of ADR symptoms for that drug by the total number of ADR reports. We calculated the incidence rate of ADR symptoms reported for each drug and performed a descriptive comparative analysis. To obtain meaningful conclusions, we categorized descriptive variables using frequencies and percentages. Statistical significance was set at a p-value of less than 0.05.

## 3 Result

### 3.1 Case description of the study

According to the WHO-VigiAccess database statistics, as of March 2025, the global ADR reports for five drugs show the following characteristics: Cetuximab (first reported in 2003) has a total of 49,527 reports, with a significant male proportion (62.59%), females accounting for 30.87%, and unknown gender making up 6.54%. The age distribution is dominated by the 45–64 years group (35.75%), followed by 65–74 years (22.79%). Regionally, the Americas account for the highest proportion (49.27%), followed by Europe (24.45%) and Asia (23.75%). Historical data shows that 38.06% of the reports were concentrated before 2015, with reports from 2024 accounting for 10.40% (5,151 cases). Pembrolizumab (first reported in 2009) has a total of 88,762 reports, with a relatively balanced gender distribution (female 44.90%, male 49.64%). The age groups are mainly 45–64 years (24.39%) and 65–74 years (22.75%), with a higher reporting rate in the elderly population (>75 years) at 15.18%. Asia is its primary reporting region (40.06%), followed by Europe (27.07%). The report volume surged in 2024, accounting for 30.44% (27,021 cases), reflecting a significant increase in safety concerns in recent years. Nivolumab (first reported in 2012) has the highest report volume (100,907 cases), with 60.61% male and 30.44% female. The 45–64 years age group accounts for 26.75%, followed by 65–74 years (24.44%). The geographic distribution is concentrated in the Americas (32.69%) and Europe (31.09%). Report volumes from 2021 to 2023 remained relatively high (8.67%–13.27%), with the 2024 report volume accounting for 22.08% (22,283 cases). Atezolizumab (first reported in 2012) has a total of 28,583 reports, with 57.70% male and 31.72% female. The 65–74 years group is the most prevalent age group (27.60%), with Asia accounting for nearly half of the reports (48.78%). The report volume for 2024 accounted for 29.40% (8,403 cases), with 2023 also showing a relatively high proportion (18.64%). Durvalumab (first reported in 2014) has the least number of reports (15,382 cases), with 58.74% male and 28.02% female. The 65–74 years group accounts for 27.29%, with Asia being the primary reporting region (47.34%). The report volume for 2024 saw a sharp increase, accounting for as high as 46.21% (7,108 cases). Table 2 presents the details.

**TABLE 2:** Five anti-HNSCC drugs adverse reports’ Demographic data.

	Cetuximab	Pembrolizumab	Nivolumab	Atezolizumab	Durvalumab
First Report Year	2003	2009	2012	2012	2014
Number of ADR reports	49,527	88,762	100,907	28,583	15,382
Female	15,289 (30.87%)	39,855 (44.90%)	30,720 (30.44%)	9067 (31.72%)	4310 (28.02%)
Male	31,001 (62.59%)	44,059 (49.64%)	61,164 (60.61%)	16,492 (57.70%)	9036 (58.74%)
Unknown	3237 (6.54%)	4848 (5.46%)	9023 (8.94%)	3024 (10.58%)	2036 (13.24%)
<18	49 (0.10%)	134 (0.15%)	277 (0.27%)	26 (0.09%)	9 (0.06%)
18–44	3219 (6.50%)	4596 (5.18%)	5528 (5.48%)	1065 (3.73%)	254 (1.65%)
45–64	17,704 (35.75%)	21,649 (24.39%)	26,996 (26.75%)	7468 (26.13%)	3333 (21.67%)
65–74	11,288 (22.79%)	20,191 (22.75%)	24,662 (24.44%)	7890 (27.60%)	4197 (27.29%)
>75	5003 (10.10%)	13,473 (15.18%)	14,259 (14.13%)	4544 (15.90%)	2238 (14.55%)
Unknown	12,264 (24.76%)	28,719 (32.36%)	29,185 (28.92%)	7590 (26.55%)	5351 (34.79%)
Africa	767 (1.55%)	1048 (1.18%)	286 (0.28%)	142 (0.50%)	151 (0.98%)
Americas	24,403 (49.27%)	26,142 (29.45%)	32,983 (32.69%)	6394 (22.37%)	3343 (21.73%)
Asia	11,763 (23.75%)	35,556 (40.06%)	33,466 (33.17%)	13,943 (48.78%)	7282 (47.34%)
Europe	12,109 (24.45%)	24,030 (27.07%)	31,377 (31.09%)	7654 (26.78%)	4201 (27.31%)
Oceania	485 (0.98%)	1986 (2.24%)	2795 (2.77%)	450 (1.57%)	405 (2.63%)
2025	756 (1.53%)	2896 (3.26%)	1189 (1.18%)	530 (1.85%)	694 (4.51%)
2024	5151 (10.40%)	27,021 (30.44%)	22,283 (22.08%)	8403 (29.40%)	7108 (46.21%)
2023	4521 (9.13%)	14,037 (15.81%)	10,358 (10.26%)	5327 (18.64%)	1713 (11.14%)
2022	4429 (8.94%)	10,419 (11.74%)	9829 (9.74%)	4384 (15.34%)	823 (5.35%)
2021	3322 (6.71%)	7326 (8.25%)	8749 (8.67%)	2766 (9.68%)	1207 (7.85%)
2020	2836 (5.73%)	5524 (6.22%)	7572 (7.50%)	2431 (8.51%)	1190 (7.74%)
2019	2567 (5.18%)	8274 (9.32%)	13,390 (13.27%)	2668 (9.33%)	1819 (11.83%)
2018	2208 (4.46%)	6688 (7.53%)	11,928 (11.82%)	1286 (4.50%)	613 (3.99%)
2017	2914 (5.88%)	3930 (4.43%)	9528 (9.44%)	679 (2.38%)	148 (0.96%)
2016	1972 (3.98%)	1555 (1.75%)	4840 (4.80%)	75 (0.26%)	51 (0.33%)
Before 2015	18,851 (38.06%)	1092 (1.23%)	1241 (1.23%)	34 (0.12%)	16 (0.10%)

### 3.2 Distribution tables of 27 SOCs for five anti-HNSCC drugs

As delineated in Table 3, the ADR reporting rates varied markedly across SOCs for the five anti-HNSCC drugs: Cetuximab, Pembrolizumab, Nivolumab, Atezolizumab, and Durvalumab. Cetuximab exhibited the highest reporting rate for skin and subcutaneous tissue disorders (20.88%), significantly exceeding other agents (Pembrolizumab: 6.05%; Nivolumab: 6.76%; Atezolizumab: 5.21%; Durvalumab: 4.76%). In contrast, Durvalumab demonstrated a disproportionately elevated incidence of respiratory, thoracic, and mediastinal disorders (18.53%), which was 2.2–3.5-fold higher than other drugs (Cetuximab: 6.26%; Pembrolizumab: 7.31%; Nivolumab: 8.28%; Atezolizumab: 7.89%). Pembrolizumab, Nivolumab, and Atezolizumab are associated with higher rates of systemic and administration site diseases (14.22%, 14.86%, and 17.90%, respectively). In the SOC of gastrointestinal disorders, ADRs were relatively high: cetuximab (11.23%), pembrolizumab (9.35%), nivolumab (10.98%), atezolizumab (10.36%), and durvalumab (8.62%). These findings underscore distinct toxicity patterns among the agents.

**TABLE 3:** Cetuximab, Pembrolizumab, Nivolumab, Atezolizumab, and Durvalumab’s report rates for 27 SOCs.

System organ class	Cetuximab	Pembrolizumab	Nivolumab	Atezolizumab	Durvalumab
Blood and lymphatic system disorders	4009 (3.62%)	6678 (3.30%)	5527 (2.57%)	3240 (5.51%)	1279 (4.65%)
Cardiac disorders	2021 (1.82%)	4453 (2.20%)	4790 (2.23%)	1288 (2.19%)	619 (2.25%)
Congenital, familial, and genetic disorders	30 (0.03%)	79 (0.04%)	58 (0.03%)	22 (0.04%)	16 (0.06%)
Ear and labyrinth disorders	175 (0.16%)	342 (0.17%)	525 (0.24%)	122 (0.21%)	52 (0.19%)
Endocrine disorders	61 (0.06%)	8544 (4.22%)	12,527 (5.83%)	1699 (2.89%)	901 (3.28%)
Eye disorders	1083 (0.98%)	2177 (1.08%)	2518 (1.17%)	421 (0.72%)	221 (0.80%)
Gastrointestinal disorders	12,444 (11.23%)	18,912 (9.35%)	23,603 (10.98%)	6087 (10.36%)	2371 (8.62%)
General disorders and administration site conditions	14,449 (13.04%)	28,775 (14.22%)	31,947 (14.86%)	10,520 (17.90%)	3726 (13.55%)
Hepatobiliary disorders	626 (0.57%)	4624 (2.29%)	6643 (3.09%)	1755 (2.99%)	739 (2.69%)
Immune system disorders	3465 (3.13%)	1644 (0.81%)	1735 (0.81%)	526 (0.90%)	185 (0.67%)
Infections and infestations	5977 (5.39%)	8832 (4.37%)	10,844 (5.04%)	3535 (6.01%)	1497 (5.44%)
Injury, poisoning and procedural complications	10,551 (9.52%)	18,039 (8.92%)	14,102 (6.56%)	3877 (6.60%)	1681 (6.11%)
Investigations	5978 (5.39%)	13,590 (6.72%)	11,888 (5.53%)	4551 (7.74%)	1917 (6.97%)
Metabolism and nutrition disorders	4221 (3.81%)	7316 (3.62%)	8994 (4.18%)	2468 (4.20%)	776 (2.82%)
Musculoskeletal and connective tissue disorders	1393 (1.26%)	8362 (4.13%)	10,222 (4.75%)	2155 (3.67%)	1051 (3.82%)
Neoplasms benign, malignant and unspecified (incl cysts and polyps)	3042 (2.74%)	14,634 (7.23%)	13,269 (6.17%)	1541 (2.62%)	1732 (6.30%)
Nervous system disorders	4429 (4.00%)	10,178 (5.03%)	10,940 (5.09%)	2860 (4.87%)	1111 (4.04%)
Pregnancy, puerperium and perinatal conditions	6 (0.01%)	31 (0.02%)	96 (0.05%)	9 (0.02%)	1 (0.00%)
Psychiatric disorders	934 (0.84%)	2824 (1.40%)	2376 (1.11%)	568 (0.97%)	278 (1.01%)
Renal and urinary disorders	1085 (0.98%)	5331 (2.63%)	5144 (2.39%)	1934 (3.29%)	442 (1.61%)
Reproductive system and breast disorders	166 (0.15%)	530 (0.26%)	360 (0.17%)	94 (0.16%)	43 (0.16%)
Respiratory, thoracic and mediastinal disorders	6937 (6.26%)	14,799 (7.31%)	17,807 (8.28%)	4635 (7.89%)	5097 (18.53%)
Skin and subcutaneous tissue disorders	23,146 (20.88%)	12,239 (6.05%)	14,529 (6.76%)	3064 (5.21%)	1308 (4.76%)
Social circumstances	72 (0.07%)	585 (0.29%)	175 (0.08%)	20 (0.03%)	20 (0.07%)
Surgical and medical procedures	943 (0.85%)	4645 (2.30%)	989 (0.46%)	112 (0.19%)	67 (0.24%)
Vascular disorders	3530 (3.19%)	3678 (1.82%)	3219 (1.50%)	1635 (2.78%)	348 (1.27%)
Product issues	69 (0.06%)	513 (0.25%)	190 (0.09%)	42 (0.07%)	25 (0.09%)

### 3.3 The most common adverse reactions of five anti-HNSCC drugs

The ADR profiles of five anti-HNSCC drugs—cetuximab, pembrolizumab, nivolumab, atezolizumab, and durvalumab—were analyzed using the WHO-VigiAccess database, revealing distinct toxicity patterns across SOCs.Table 4 lists the 20 most frequently reported adverse reactions for the five anti-HNSCC drugs, presented as preferred terms within the SOCs. Cetuximab exhibited a predominant dermal toxicity profile, with rash (5.74%) and off-label use (5.98%) as the most frequently reported ADRs. Cutaneous events, including pruritus (2.21%), acne (1.87%), and erythema (1.20%), collectively accounted for 12.45% of reports. Pembrolizumab demonstrated a higher incidence of immune-related adverse events (irAEs), notably malignant neoplasm progression (5.56%). Nivolumab shared similar irAE patterns, with malignant neoplasm progression (4.23%). It is worth noting that Atezolizumab showed outstanding Off-label use (3.99%) and Death (3.20%). Durvalumab displayed a unique safety signal: pneumonitis (8.28%) and radiation pneumonitis (2.44%) were reported at rates 2.2–8.2-fold higher than other agents (nivolumab: 1.22%; pembrolizumab: 1.01%).

**TABLE 4:** Top 20 adverse reactions for five anti-HNSCC drugs.

Cetuximab		Pembrolizumab		Nivolumab		Atezolizumab		Durvalumab	
ADR	Report rate	ADR	Report rate	ADR	Report rate	ADR	Report rate	ADR	Report rate
Off label use	5.98%	Malignant neoplasm progression	5.56%	Malignant neoplasm progression	4.23%	Off label use	3.99%	Pneumonitis	8.28%
Rash	5.74%	Death	2.44%	Death	4.03%	Death	3.20%	Death	4.29%
Death	2.21%	Inappropriate schedule of product administration	2.33%	Diarrhoea	2.36%	No adverse event	2.78%	Malignant neoplasm progression	3.11%
Pruritus	2.21%	Fatigue	1.78%	Interstitial lung disease	1.90%	Pyrexia	1.89%	Interstitial lung disease	2.98%
Diarrhoea	2.14%	Diarrhoea	1.71%	Off label use	1.84%	Disease progression	1.72%	Radiation pneumonitis	2.44%
Acne	1.87%	Interstitial lung disease	1.67%	Fatigue	1.76%	Diarrhoea	1.71%	Diarrhoea	1.72%
Nausea	1.83%	Hypothyroidism	1.58%	Hypothyroidism	1.61%	Fatigue	1.64%	Dyspnoea	1.59%
Dyspnoea	1.77%	Product use in unapproved indication	1.56%	Intentional product use issue	1.52%	Asthenia	1.36%	Pneumonia	1.51%
Infusion related reaction	1.62%	Off label use	1.50%	Pyrexia	1.52%	Interstitial lung disease	1.30%	Off label use	1.44%
Vomiting	1.49%	Rash	1.25%	Rash	1.45%	Pneumonitis	1.26%	Fatigue	1.43%
Malignant neoplasm progression	1.36%	Product use issue	1.19%	Colitis	1.37%	Pneumonia	1.22%	Asthenia	1.32%
Dermatitis acneiform	1.32%	Nausea	1.17%	Asthenia	1.24%	Dyspnoea	1.22%	Pyrexia	1.25%
Pyrexia	1.24%	Pyrexia	1.09%	Pneumonitis	1.22%	Anaemia	1.21%	Rash	1.16%
Erythema	1.20%	Asthenia	1.09%	Pruritus	1.21%	Decreased appetite	1.18%	Hypothyroidism	1.05%
Dry skin	1.18%	Pneumonia	1.02%	Adrenal insufficiency	1.18%	Nausea	1.15%	Pruritus	1.02%
Hypotension	1.11%	Pneumonitis	1.01%	Dyspnoea	1.17%	Rash	1.15%	Cough	1.01%
Neutropenia	1.04%	Dyspnoea	0.92%	Nausea	1.16%	Febrile neutropenia	1.08%	Colitis	0.99%
Hypersensitivity	1.02%	Decreased appetite	0.91%	Decreased appetite	1.00%	Hypertension	1.08%	Nausea	0.92%
Asthenia	0.99%	Pruritus	0.87%	Pneumonia	0.97%	Neutropenia	1.02%	Anaemia	0.87%
Disease progression	0.97%	Drug ineffective	0.85%	Arthralgia	0.82%	Pruritus	1.01%	Febrile neutropenia	0.84%

### 3.4 Commonalities in the most common adverse reactions of five anti-HNSCC drugs

As delineated in Table 5, The analysis of common adverse reactions across five anti-HNSCC agents (Cetuximab, Pembrolizumab, Nivolumab, atezolizumab, Durvalumab) demonstrated that systemic and administration site-related events (Signal N = 18) were the most frequently reported, primarily including disease progression, death, multi-organ dysfunction, and nonspecific symptoms (e.g., pyrexia, fatigue, and mucosal inflammation). Gastrointestinal toxicities (Signal N = 13) were prominently observed, with diarrhea, vomiting, and abdominal pain as the predominant manifestations. Laboratory abnormalities (Signal N = 11) focused on cytopenias (e.g., leukopenia and thrombocytopenia) and elevated hepatic enzymes. Cutaneous reactions (Signal N = 10), neurologic events (Signal N = 10), and respiratory disorders (Signal N = 10) manifested as rash, headache, and dyspnea, respectively, while infection-related complications (Signal N = 9) included sepsis, pneumonia, and urinary tract infections. Notably, low-frequency but severe events were identified, encompassing cardiac toxicity (e.g., myocardial infarction, Signal N = 4), acute kidney injury (Signal N = 3), and hepatic failure (Signal N = 1).

**TABLE 5:** Common adverse reactions o**f** five anti-HNSCC drugs.

System organ classes	ADRs	Signal N
Blood and lymphatic system disorders	Leukopenia, Febrile neutropenia, Thrombocytopenia,Pancytopenia, Neutropenia,Anaemia	6
Cardiac disorders	Cardiac arrest, Myocardial infarction, Atrial fibrillation, Cardiac failure, Tachycardia	4
Neoplasms benign, malignant and unspecified (incl cysts and polyps)	Neoplasm progression, Malignant neoplasm progression	2
Eye disorders	Vision blurred	1
Gastrointestinal disorders	Abdominal pain upper, Abdominal distension, Dysphagia,Diarrhoea, Vomiting,Ascites, Constipation,Colitis, Abdominal pain, Nausea,Stomatitis,Dry mouth, Dyspepsia	13
General disorders and administration site conditions	Peripheral swelling, Asthenia,Condition aggravated, Pain,Drug ineffective, Multiple organ dysfunction syndrome, Death,Chest pain, Malaise,Illness, Fatigue,Oedema peripheral, Swelling,Pyrexia, Chills,Oedema, Disease progression, General physical health deterioration, Mucosal inflammation	18
Hepatobiliary disorders	Hepatic failure	1
Immune system disorders	Hypersensitivity, Anaphylactic reaction	2
Infections and infestations	Sepsis, Infection,Nasopharyngitis, Pneumonia,Influenza, Pneumonia aspiration, Urinary tract infection, Septic shock, Cellulitis	9
Injury, poisoning and procedural complications	Toxicity to various agents,Off label use,Infusion related reaction, Fall,Product use in unapproved indication	5
Investigations	Weight decreased, Aspartate aminotransferase increased, Blood creatinine increased, Platelet count decreased, Oxygen saturation decreased, Haemoglobin decreased, Neutrophil count decreased, Alanine aminotransferase increased, Blood alkaline phosphatase increased, Blood bilirubin increased, White blood cell count decreased	11
Metabolism and nutrition disorders	Dehydration, Hypokalaemia,Hyperglycaemia, Decreased appetite, Hyponatraemia,Hyperkalaemia	6
Musculoskeletal and connective tissue disorders	Neck pain, Muscular weakness, Muscle spasms, Back pain, Arthralgia,Pain in extremity, Myalgia	7
Skin and subcutaneous tissue disorders	Hyperhidrosis, Skin toxicity, Rash,Erythema,Dry skin, Dermatitis,Urticaria, Alopecia,Pruritus, Rash pruritic, Skin disorder	10
Nervous system disorders	Headache, Paraesthesia,Dizziness, Tremor,Somnolence, Hypoaesthesia,Syncope, Cerebrovascular accident, Seizure,Neuropathy peripheral	10
Psychiatric disorders	Confusional state, Insomnia,Anxiety	3
Renal and urinary disorders	Acute kidney injury, Renal impairment, Renal failure	3
Respiratory, thoracic and mediastinal disorders	Respiratory failure, Oropharyngeal pain, Interstitial lung disease, Pneumothorax,Dyspnoea, Cough,Pulmonary embolism, Haemoptysis,Pleural effusion, Dysphonia,Hypoxia, Pneumonitis	10
Vascular disorders	Thrombosis, Hypotension,Deep vein thrombosis, Flushing,Haemorrhage, Hypertension	6

### 3.5 Disproportionality analysis

**FIGURE 1:**
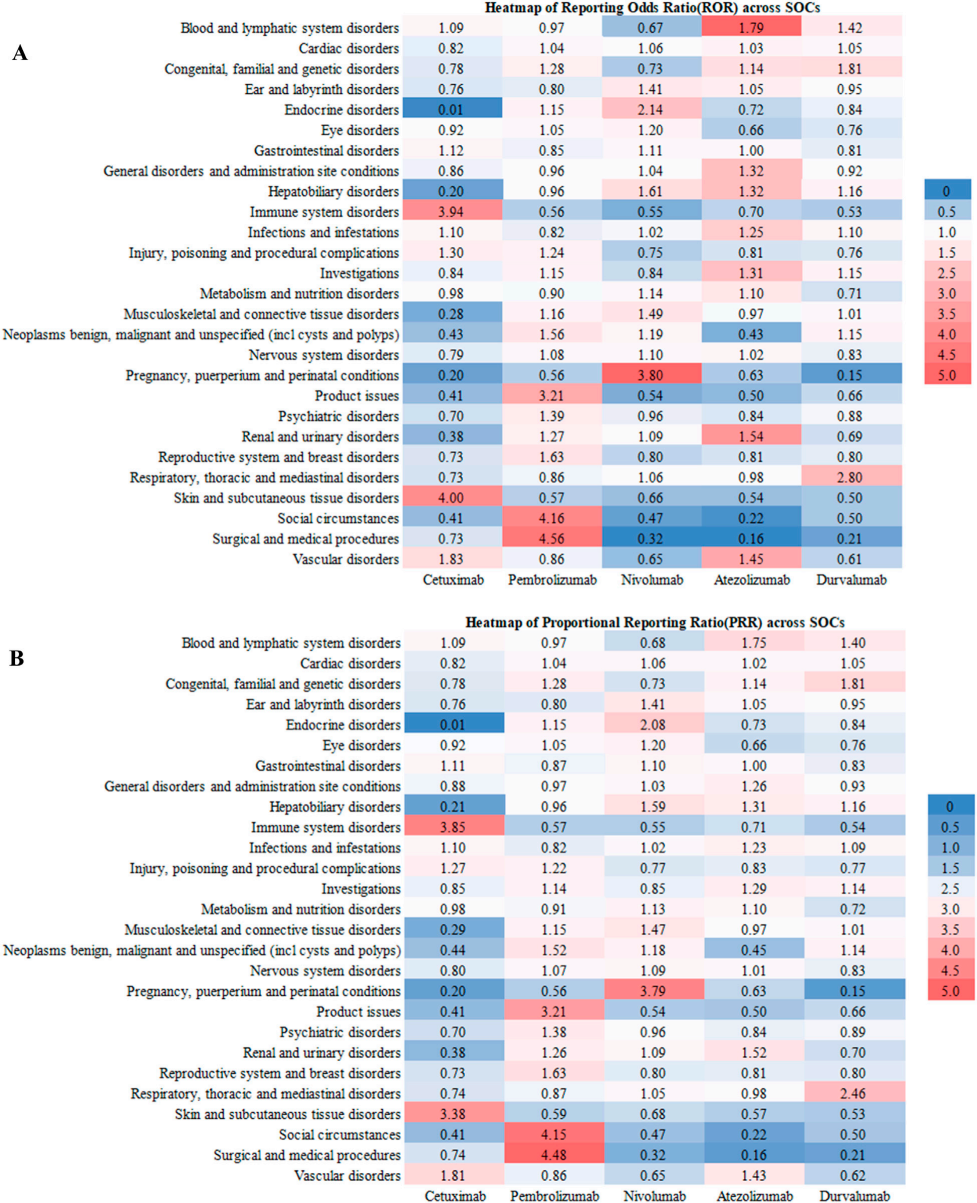
The RoR **(A)** and PRR **(B)** heatmap illustrates the safety profiles of the five anti-HNSCC drgus across different SOCs, highlighting specific areas of elevated risk for each drug.

As delineated in Figure 1, The risk stratification analysis based on the SOC reveals the unique safety characteristics of different immunotherapy drugs, specifically as follows: Cetuximab shows significant risk signals in immune system diseases (RoR = 3.94; PRR = 3.85), with its risk level far exceeding the other four drugs. Additionally, its high-risk features in the skin and subcutaneous tissue diseases (RoR = 4.00; PRR = 3.38) suggest that it may cause severe skin toxicity (such as rashes or mucositis). Pembrolizumab’s main risks are concentrated in complications related to surgery and medical procedures (RoR = 4.56; PRR = 4.48), with its risk intensity being 4–5 times that of other systems. Nivolumab’s reproductive system risk features are particularly distinctive, with abnormal increases in risk signals in pregnancy-related diseases (RoR = 3.80; PRR = 3.79). Atezolizumab’s most prominent risks are in the blood and lymphatic systems (RoR = 1.79; PRR = 1.75), possibly increasing the risk of anemia or thrombocytopenia. Durvalumab’s respiratory system toxicity is significantly higher than that of other drugs (RoR = 2.80; PRR = 2.46), necessitating caution regarding the risk of interstitial pneumonia. Furthermore, its higher risk in congenital diseases (RoR = 1.81; PRR = 1.81) suggests that potential genetic toxicity requires further investigation.

### 3.6 Serious adverse events of five anti-HNSCC drugs

**FIGURE 2:**
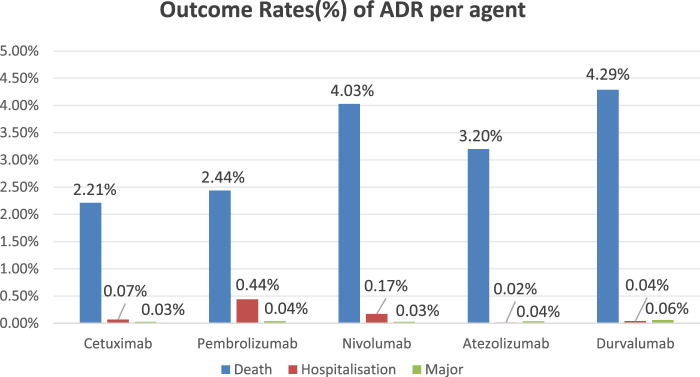
Major adverse event rates for five anti-HNSCC drgus.

Incidence of severe adverse events (including mortality, hospitalization, and life-threatening incidents) among five anti-HNSCC agents: Cetuximab: Mortality (2.21%), Hospitalization (0.07%), Major Events (0.03%); Pembrolizumab: Mortality (2.44%), Hospitalization (0.44%), Major Events (0.04%); Nivolumab: Mortality (4.03%), Hospitalization (0.17%), Major Events (0.03%); Atezolizumab: Mortality (3.20%), Hospitalization (0.02%), Major Events (0.04%); Durvalumab: Mortality (4.29%), Hospitalization (0.04%), Major Events (0.06%). The bar chart demonstrates comparative incidence rates of primary adverse events across therapeutic agents (Figure 2).

## 4 Discussion

The global health burden of HNSCC continues to rise, particularly among high-risk groups associated with smoking and alcohol consumption. If left untreated, HNSCC can lead to severe health consequences, including death (Chintala et al., 2022).In recent years, novel immunotherapies and targeted treatments have offered more options for patients, but the potential ADRs of these therapies remain a major challenge in clinical application.his study analyzes data from the WHO-VigiAccess database, with a particular focus on ADRs associated with five promising anti-HNSCC drugs—cetuximab, pembrolizumab, nivolumab, atezolizumab, and durvalumab—highlighting their overall ADR profiles, distribution across different SOCs, and disproportionality in immune-related ADRs.The results show that different drugs exhibit significantly heterogeneous safety profiles due to differences in mechanisms of action and indications, underscoring the need for personalized monitoring strategies. These findings not only reveal the challenges in anti-HNSCC drug therapies but also provide important reference points for clinical practice.

Global ADR data analysis reveals a significant number of ADR reports associated with these five anti-HNSCC drugs, totaling 145,678 reports. In terms of gender distribution, ADRs reported by male patients are dominant, which could be linked to male patients’ treatment needs and pharmacokinetic differences. Studies have shown that male patients often experience more significant treatment responses and ADRs than female patients across many types of cancer, possibly due to differences in hormone levels, immune responses, and drug metabolism (Dai et al., 2025). For example, males generally have a higher drug clearance rate, which may lead to fluctuations in drug concentrations in the body, thereby increasing the risk of ADRs (Venturini et al., 2011). For instance, the activity of certain cytochrome P450 enzymes might be higher in males than in females, accelerating drug metabolism and thereby affecting drug efficacy (Tran et al., 1998). Additionally, smoking and alcohol consumption are major risk factors for HNSCC, with males typically having higher rates of these behaviors than females (Johnson et al., 2020). These lifestyle factors may influence drug metabolism and efficacy, increasing the risk of ADRs.The age distribution shows that the 45–64 age group has the highest proportion of ADRs. Patients in this age group are typically middle-aged and elderly, often with comorbid conditions, and long-term exposure to carcinogenic environmental factors (such as smoking and drinking) further increases their risk of HNSCC. Elderly populations, in particular, are more likely to experience immune-related adverse events when undergoing immune checkpoint inhibitor therapy, which is associated with age-related immune system decline and enhanced autoimmune responses (Wang et al., 2021). Additionally, older patients may experience more drug interactions due to comorbidities or polypharmacy, thereby increasing the risk of ADRs (Yadesa et al., 2021). The immune system ages with increasing age, leading to a decline in immune function, which may affect the efficacy and toxicity of immune checkpoint inhibitors (Baik et al., 2017). Furthermore, geographic distribution data show that the highest ADR reports come from the Americas and Europe. This phenomenon may reflect differences in drug availability, healthcare systems, and pharmacovigilance practices. The disparities in drug accessibility across regions may lead to patients being exposed to different treatment regimens, which can influence ADR reporting. For example, in the Americas and Europe, regulatory systems for drugs are relatively well-established, and pharmacovigilance measures are effectively implemented, resulting in a higher number of ADR reports (Valinciute-Jankauskiene and Kubiliene, 2021). In contrast, in some low-income countries and regions, the limited availability of drugs and insufficient resources may lead to fewer ADR reports, potentially underestimating the actual incidence (Onyije et al., 2024). These geographic differences and patient population characteristics provide a more comprehensive understanding of the safety and tolerability of anti-HNSCC drugs, helping further optimize treatment strategies and pharmacovigilance practices.

The five anti-HNSCC drugs evaluated in this study exhibit distinct safety profiles influenced by their pharmacological mechanisms, treatment settings, and patient characteristics. Cetuximab, as an EGFR inhibitor, has been used for a long time in the treatment of HNSCC (Elmusrati et al., 2021). It can be used alone or in combination with chemotherapy drugs to enhance treatment efficacy, especially for tumors with high EGFR expression (Pirker, 2015). However, despite its good effectiveness in treating HNSCC, Cetuximab’s ADRs related to skin and subcutaneous tissue diseases account for as much as 20.88%, mainly manifested as rash (5.74%), pruritus (2.21%), and acneiform dermatitis (1.87%). Skin toxicities such as rash and mucositis are common treatment-related side effects and typically manifest as rashes on the face, neck, and upper chest, closely related to the pharmacological effects of the drug (Puthenpurail et al., 2021). These ADRs are closely related to the abnormal differentiation of keratinocytes caused by EGFR signaling blockade, reflecting the typical skin toxicity of EGFR inhibitors (Nowaczyk et al., 2023). By inhibiting the EGFR signaling pathway, Cetuximab blocks the proliferation and repair of these cells, leading to skin cell damage and adverse reactions (Parikh et al., 2014). Clinically, preventive use of moisturizers and close monitoring of skin reactions is essential. Pembrolizumab, as a PD-1 inhibitor, blocks the interaction between PD-1 and its ligand PD-L1, restoring T-cell function and enhancing the immune system’s ability to recognize and eliminate cancer cells (Gu et al., 2024). ICIs—including Pembrolizumab, Nivolumab, Atezolizumab, and Durvalumab—achieve anti-tumor effects by enhancing T-cell activity through PD-1/PD-L1 pathway inhibition (Zhong et al., 2020; Tekiki et al., 2021; Schomberg, 2019). In this study, gastrointestinal disorders (GI) emerged as a common category of ADRs across all five drugs. The relatively high prevalence of GI-related ADRs is consistent with known toxicities such as mucositis, diarrhea, colitis, and nausea. For Cetuximab, mucositis and diarrhea may result from EGFR inhibition in the GI epithelium, which impairs mucosal repair and absorption (Hintelmann et al., 2020). For ICIs, colitis and diarrhea are well-documented irAEs resulting from loss of immune tolerance in the intestinal mucosa, likely mediated by T-cell overactivation and cytokine release (Lau et al., 2021). In addition to GI toxicity, Pembrolizumab was linked to elevated risks of surgical and medical complications (RoR: 4.56; PRR: 4.48), possibly due to its impact on wound healing and infection control in the post-surgical setting (Xu et al., 2023). Nivolumab was associated with a significantly increased risk of pregnancy-related disorders (RoR: 3.80; PRR: 3.79), suggesting potential disruption of maternal-fetal immune tolerance via enhanced T-cell activity. Atezolizumab demonstrated a notable risk in the hematologic system, with 5.51% of ADRs affecting blood and lymphatic tissues (RoR: 1.79; PRR: 1.75). This may reflect immune-mediated bone marrow suppression or autoimmunity targeting hematopoietic cells (Falette Puisieux et al., 2022). Immune checkpoint inhibitors may activate autoimmune responses, leading to attacks on normal blood cells, especially when the patient’s immune function is activated, causing immune cells to mistakenly attack normal hematopoietic tissue or blood cells, leading to hematologic adverse reactions (Zhang et al., 2021). Durvalumab, in contrast, showed the highest respiratory system toxicity (18.53%), primarily pneumonia (8.28%) and radiation pneumonitis (2.44%). These effects are likely enhanced by its use in post-chemoradiation consolidation therapy for NSCLC, where radiotherapy exacerbates lung tissue susceptibility. Routine pulmonary evaluation and radiographic monitoring are critical during treatment. Finally, both Pembrolizumab and Nivolumab—commonly used in advanced or refractory HNSCC—showed the highest rates of malignant neoplasm progression reports (Pembrolizumab: 5.83%, Nivolumab: 4.23%). This may reflect the drugs’ widespread use in late-stage disease, where tumor immune escape mechanisms can evolve in response to prolonged immune activation. Immune reprogramming of the tumor microenvironment might enable cancer cells to resist immune surveillance and promote progression or metastasis.

ICIs have demonstrated significant efficacy in the treatment of various malignancies, particularly HNSCC. The occurrence of specific irAEs may be related to the expression patterns of immune checkpoints and the immunological microenvironment in affected organs. For example, PD-1/PD-L1 expression in pulmonary tissue may lead to excessive T-cell activation, resulting in pneumonitis (Ebinama et al., 2023). Similarly, in the gastrointestinal tract, ICIs may disrupt immune tolerance and induce colitis. Endocrine organs are also susceptible, with irAEs manifesting as thyroiditis, hypophysitis, or type 1 diabetes (Takada et al., 2020). Pre-existing immune status may influence the likelihood of irAEs. Elevated levels of autoantibodies or inflammatory cytokines have been associated with increased risk (Basnet et al., 2024). In addition, the gut microbiome plays a crucial role in regulating immune responses. Studies have indicated that specific microbial compositions may be linked to irAE risk. For instance, the presence of certain bacterial strains may enhance immune activation and thereby increase susceptibility to irAEs (Naqash et al., 2021).

Although the overall incidence of SAEs—including mortality, hospitalization, and life-threatening complications—was relatively low across the five agents, their clinical significance should not be underestimated. Each drug exhibited a distinct adverse event profile. Previous studies have suggested that the risk of SAEs may be influenced by cumulative dosage and duration of treatment (Llopis-Salvia et al., 2010). However, there is currently a lack of robust data analyzing the specific causes leading to SAE outcomes. Therefore, it is essential to implement effective monitoring strategies in clinical practice. These include early recognition of symptoms and timely administration of immunosuppressive agents such as corticosteroids when indicated. Furthermore, stratifying patients based on comorbidities, PD-L1 expression, and prior treatment history may help reduce the likelihood of severe complications and improve treatment safety.

This study is limited by the inherent biases of spontaneous reporting systems. First, underreporting may disproportionately affect lower-grade toxicities, potentially underestimating their true incidence. Secondly, the lack of clinical variables such as treatment duration and dosing plan can hinder risk stratification and confuse ADR attribution. To address these limitations, future research should integrate existing adverse reaction reporting systems with hospital electronic medical record systems for analysis, to capture underreported low-level ADRs and clinical confounding factors. Additionally, the pharmacovigilance database lacked consistent data on whether ADRs occurred with monotherapy or combination regimens, limiting the assessment of each drug’s independent safety profile. Importantly, while HNSCC comprises clinically distinct subtypes (e.g., oropharyngeal, hypopharyngeal, laryngeal carcinomas) with potential variations in tumor biology and treatment response, the WHO-VigiAccess database lacks subtype-specific ADR data. Future research should integrate electronic health records and real-world evidence platforms to dynamically track the impact of dosage, treatment duration, and concomitant medications on ADRs, while also exploring the correlation between biomarkers (e.g., PD-L1 expression levels) and toxicity risks. Despite these limitations, new immunotherapies continue to evolve, demonstrating promising prospects. For instance, ongoing research on anti-PD-1 drugs and combination immunotherapies has shown high efficacy and good tolerability in early clinical trial results. While these immunotherapy drugs show broad potential for clinical application, more Phase III clinical trials and long-term safety evaluations are still needed.

### 4.1 Clinical practice recommendations

Based on the study findings, individualized management strategies should be developed for different drugs: Considering the impact of gender and age on pharmacokinetics and pharmacodynamics, individualized dosing regimens should be developed based on factors such as the patient’s gender, age, physiological functions, and comorbidities to enhance efficacy and reduce the risk of ADRs. In clinical trial design, gender and age factors should be fully considered, with stratified analysis performed to more accurately assess drug efficacy and safety. For patients receiving Cetuximab, heightened attention to dermatologic toxicity is warranted. Prophylactic skin care education, along with early intervention for rash and potential infections, is essential to manage the high incidence of cutaneous adverse events. In patients treated with ICIs such as Pembrolizumab or Nivolumab, comprehensive baseline assessments—including thyroid function, pulmonary function tests, and gastrointestinal evaluation—should be performed. Regular follow-up monitoring is recommended to promptly identify irAEs, such as thyroiditis, pneumonitis, or colitis. Prior to initiating Durvalumab therapy, pulmonary imaging should be conducted to exclude subclinical interstitial lung disease. For patients with a history of thoracic radiotherapy, extended post-treatment surveillance is advised to detect delayed-onset pulmonary complications, including radiation-induced pneumonitis. During Atezolizumab treatment, complete blood counts should be monitored regularly, with particular attention to hemoglobin levels and leukocyte differentials. Early signs of anemia or infection should be promptly addressed to ensure hematologic safety. These mechanism-driven and agent-specific strategies aim to enhance therapeutic benefit while reducing preventable ADRs. Personalized monitoring protocols guided by pharmacological risk profiles are critical for improving treatment outcomes in patients with HNSCC.

## 5 Conclusion

This study analyzed ADRs associated with five major anti-HNSCC drugs based on data from the WHO-VigiAccess database, revealing the distinct safety profiles of these drugs in treating HNSCC. Ongoing long-term safety monitoring of these drugs, along with adjustments to clinical practice based on real-world data, will be crucial for the success of future HNSCC treatments.

## Data Availability

Publicly available datasets were analyzed in this study. This data can be found here: https://www.vigiaccess.org.
